# A New Dental Disease

**Published:** 1882-01

**Authors:** N. Stevenson


### A NEW DENTAL DISEASE.

A child, aged ten, whose teeth six months ago appeared to be all perfectly sound, came to me with toothache in the right lower canine. I found that a large portion of the enamel had disappeared from the front surface of the tooth, as if it had been chipped violently off; the dentine was all exposed, but there was no softening or appearance of decay. The disease, which has commenced in several of the other incisor teeth, appears first as a small white spot in about the thickest part of the front surface of the enamel, which it seems to penetrate, and, then, suddenly disintegrating, this comes away, and exposes the remaining sensitive enamel and the dentine. This disease is altogether a different thing from the gradual decay, or wear at the neck of the teeth, frequently met with in adults, for in this case the patient is only ten, and, as far as I have been able to ascertain, the incisors and canines never have been known to decay in the manner above described. We are often at our wits' end to cope with the increasing prevalence of caries in the teeth of the very young; and if this be (as I fear it is) a new form of destructive energy, the sooner it is recognized the better.—*N. Stevenson, M. R. C. S., in British Medical Journal.*

